# Phage Renaissance: New Hope against Antibiotic Resistance

**DOI:** 10.1289/ehp.121-a48

**Published:** 2013-02-01

**Authors:** Carol Potera

**Affiliations:** **Carol Potera**, based in Montana, has written for *EHP* since 1996. She also writes for *Microbe*, *Genetic Engineering News*, and the *American Journal of Nursing*.

In the 1920s and 1930s, before widespread use of anti-biotics, physicians successfully treated a variety of infections with bacteriophages, or phages for short. These natural viral predators, which target bacteria but leave mammalian and plant cells unscathed, were sold by pharmaceutical companies including Eli Lilly & Company^1^ and even made it into the fiction of the time—the protagonist of Sinclair Lewis’ 1925 book *Arrowsmith* fought bubonic plague with phages.^2^

**Figure:**
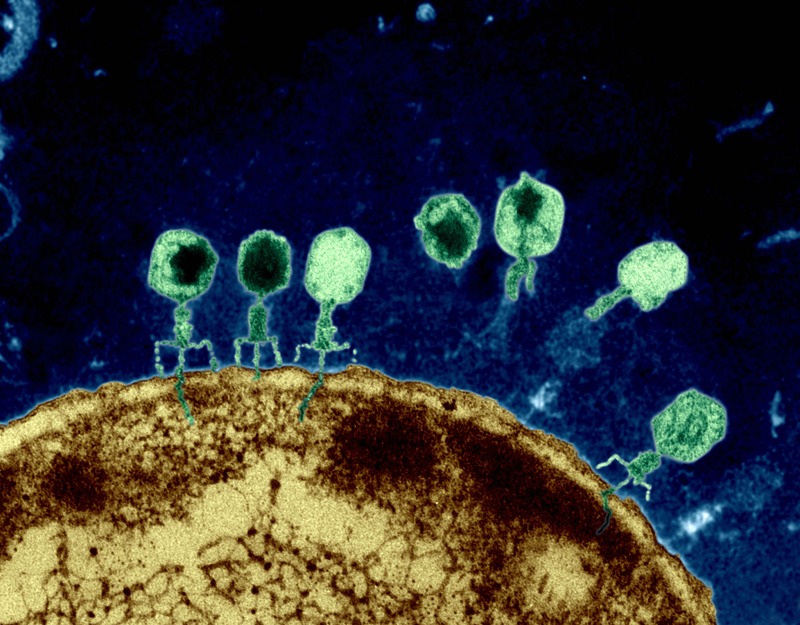
Phages on the surface of an Escherichia coli cell inject genetic material into the bacterium. © Eye of Science/Science Source

Scientists of the day did not understand exactly how phages killed bacteria, and their crude therapies performed inconsistently. So with the mass production of “magic bullet” antibiotics in the 1940s and 1950s, interest in phages largely waned.^1^

But 60 years later, antibiotics are losing their luster. Antibiotics have not only been used to treat human infections but also are given to farm animals to speed growth and prevent illness. They end up being flushed down drains and leach into soil and groundwater, where they contribute to environmental hot spots of antibiotic resistance.^3^ Antibiotic-resistant microbes now pose a growing threat to people of all ages, nationalities, and socioeconomic backgrounds, and previously treatable diseases are becoming untreatable once more.^4^

Researchers, too, are looking back to the pre-antibiotic era, but with the goal of resurrecting phages as antidotes for antibiotic resistance and solving medical, agricultural, and environmental problems. This time around, they are armed with molecular biology tools to better understand and control phages. They can also draw on the experience of Eastern European investigators, who have continued to study these viruses for decades, publishing their work primarily in Russian, Georgian, and Polish journals.^1^ In these countries, phages have continued to be given orally as tablets and liquids, topically, rectally, and as injections for 90 years. No reports of serious side effects have been recorded, and fever and other minor side effects came from contaminants like endotoxins in early phage preparations.^1^

## Cocktails for Food Safety

Foodborne illness is a serious problem, causing 9.4 million cases of foodborne disease, nearly 56,000 hospitalizations, and more than 1,350 deaths yearly in the United States alone, according to estimates from the Centers for Disease Control and Prevention.^5^ These illnesses are the target of phage therapies produced by biotech development company Intralytix, Inc.^6^

Intralytix cofounder and chief scientific officer Alexander Sulakvelidze remembers listening to colleague Glenn Morris, a physician at the University of Maryland School of Medicine in Baltimore, vent his frustrations when multidrug-resistant infections started claiming the lives of his patients in the mid-1990s. Sulakvelidze, then a visiting researcher from Georgia in the former Soviet Union, was stunned that Morris had never heard of phages. “It was like lightning struck,” he says of learning that phages were not used in the United States, despite all the country’s advanced medical technologies. “People die because western medicine is no longer aware of phage therapy.” Together, Sulakvelidze and Morris launched Intralytix in 1998.

Today the company sells two phage products approved by the U.S. Food and Drug Administration (FDA) and U.S. Department of Agriculture (USDA). Each product contains a “cocktail” of phages that target and kill the same bacterium. “Our technology goes after only bad bacteria,” says Sulakvelidze. By comparison, he says, antibiotics and chemical disinfectants also kill good bacteria “like casualties of war.” There’s an increasing recognition that we cannot kill all microbes, “and we don’t want to,” Sulakvelidze notes, “because without bacteria there would be no life on Earth.”

Intralytix’s ListShield™, the first phage product approved by the FDA as a food additive, targets *Listeria monocytogenes* in ready-to-eat meat and poultry (e.g., deli meats and frankfurters).^7^ The microbe, which also contaminates dairy products and raw produce, grows even in refrigerated foods and causes a serious infection called listeriosis with a fatality rate of about 20%.^5^

Eliminating the bacterium from food processing plants is very difficult. “Despite intense cleaning by food processors, *L. monocytogenes* is ubiquitous and stubborn,” says Sulakvelidze. ListShield is sprayed on meat products as well as on drains, floors, coolers, and other surfaces that might harbor *L. monocytogenes* at food processing plants. According to Sulakvelidze, the product typically reduces *L. monocytogenes* contamination by 95% or better.

A second product, EcoShield™, is sprayed on red meat before grinding into hamburger to kill *Escherichia coli* O157:H7, the cause of 62,000 foodborne diseases yearly in the United States.^5^ Meat trimmings from different carcasses are combined into ground meat, and bacteria from just one animal can infect large batches of meat. In studies with government investigators, Sulakvelidze demonstrated that EcoShield killed 95–100% of *E. coli* O157:H7 within 5 minutes.^8^

Jitu Patel and Manan Sharma, researchers at the USDA in Beltsville, Maryland, have also tested EcoShield on fresh-cut lettuce and cantaloupes experimentally contam-inated with *E. coli* O157:H7. Biofilms, or persistent colonies, of this pathogen can contaminate blades used to harvest lettuce, spinach, and other crops. Even though blades are disinfected with chlorine, some of the cells in a biofilm may elude killing. In the USDA studies, however, EcoShield reduced pathogen levels by 100-fold within a day.^9^ Sharma says he knows of no one using the product to treat fruits or vegetables.

EcoShield and ListShield are odorless, tasteless, invisible, and noncorrosive. The phages in these products are present at 0.001% in a liquid spray, making the final solution nearly “as benign as water to anything except targeted bacteria,” according to Sulakvelidze. The phages quickly dissipate, and no phage solution residue is passed on to consumers.

A third product, SalmoFresh™, is pending FDA approval and will target *Salmonella* in poultry and other foods. Intralytix is working on other phage treatments for wound healing, veterinary care, and oral health.

## Lysins: An Alternative Approach

As an alternative to phage cocktails, some researchers are isolating the phage enzymes that make bacteria explode (see box “How Phages Work”). When a phage replicates within a host bacterial cell, two key enzymes are produced—holins, which perforate the inner cell membrane, and lysins, which enter through the holes created by the holins and attack the cell wall, eventually bursting the cell like a balloon to release hundreds more phages.^10^

Significantly, says Daniel Nelson, an assistant professor at the University of Maryland’s Institute for Bioscience and Biotechnology Research, lysins applied directly to bacteria can “chew up” and destroy the cell wall from the outside even in the absence of phages or holins. “It’s called ‘lysis from without,’ meaning lysis without phage infection,” Nelson explains.

**Figure:**
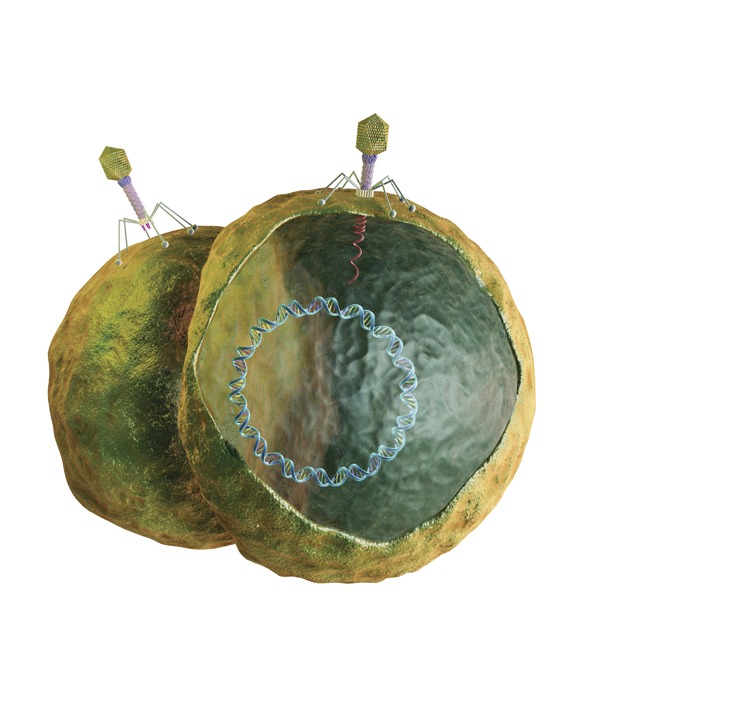
How Phages Work All known bacteria are thwarted by phages, which are extremely specific and only attack the strain of bacteria they evolved to inhabit and kill (mammalian and plant cells lack the receptors required for phage infection, so phages are harmless against them). Phages first attach to and puncture the bacterial membrane. Phage DNA is injected into the host cell. The host cell’s DNA transcription is suppressed, and phage-specific proteins are synthesized instead. New phages are assembled, the host cell membrane is disrupted, and large numbers of new phages are released from the host bacterium, which dies.^22^ An estimated 10^30^–10^32^ phages exist in the biosphere,^22^ and an estimated 10^23^ phage infections occur per second.^24^ Every 48 hours, phages destroy about half the bacteria in the world,^25^,^26^ a dynamic process that occurs in all ecosystems.^14^,^24^ Phages have infected bacteria for billions of years, and just as bacteria mutate to resist drugs, they also mutate to render phages ineffective. However, new phages continually evolve against the mutated bacteria.^27^ “It’s an evolutionary arms race,” says Daniel Nelson of the University of Maryland’s Institute for Bioscience and Biotechnology Research. Because phages cannot reproduce on their own, they must infect bacteria, which, in turn, spend massive amounts of energy trying to avoid death by phage. However, phages are not totally bad and even offer bacteria a fitness advantage by transferring genes for antibiotic resistance and toxins to bacteria. To acquire desirable traits while avoiding death, bacteria use restriction modification systems to cut out deleterious phage DNA and keep beneficial phage DNA.^27^ “Nonetheless, phages adapt and evolve more rapidly than bacteria, so the cat-and-mouse game continues as both sides try to out-evolve each other,” says Nelson. © Medi-Mation Ltd/Science Source

Lysins are being used in the fight against *Clostridium perfringens*, which causes necrotic enteritis in poultry and also is a leading cause of food poisoning in people.^11^ “We want to protect chickens and poultry products eaten by people with phage products,” says Bruce Seal, a microbiologist and research leader at the USDA’s Agricultural Research Service in Athens, Georgia.

Seal and colleagues have identified more than 50 strains of *C. perfringens* at poultry production sites, each with its own specific phage. “We would need cocktails with twenty to thirty phages to kill all the *C. perfringens* strains we find just in the Southeastern United States,” Seal says. Instead, his team combines different lysins that all kill *C. perfringens* using a variety of tools, such as amidases, peptidases, and lysozymes.^12^ Peptidases break down proteins, amidases break amide bonds, and lysozymes break down peptidoglycans found in cell walls of bacteria. Combined, they are more effective than any single approach alone.

Most *Clostridium* species help chickens digest feed and provide nutritional factors to the birds, so it’s important to kill only the pathogenic *C. perfringens*, says Seal. The multi-lysin approach may not only prove more effective now but also deter bacterial resistance in the future. There are no known reports of lysin resistance, but “it could happen,” says Seal. He says lysins haven’t been used this way long enough for people to know whether resistance will occur. There are many lysins to choose from, he adds, and it seems logical to assume new ones could be found when/if needed.

## Medical Applications

Phages are also now being explored in medical applications in the United States. In 2008 the FDA approved the first phase 1 clinical trial to evaluate an unnamed cocktail of eight phages prepared by Intralytix to treat venous leg ulcers. The phages target *Pseudomonas aeruginosa*, *Staphylococcus aureus*, and *E. coli*. The treatment was proven safe, the main goal of phase 1 trials.^13^ “Before this, no formal study was ever done in the United States to show the safety of phage cocktails,” says Sulakvelidze. Funding is needed to proceed to phase 2 trials to evaluate how well the treatment works.

Nelson and colleagues at Rockefeller University in New York City have purified lysins that in animal studies killed *Streptococci* responsible for scarlet fever, rheumatic fever, necrotizing fasciitis (“flesh-eating disease”), and pneumonia.^10^^,^^14^ They recently solved the X-ray crystal structure of PlyC, the most powerful lysin known, providing clues to its superior potency.^15^ PlyC is 100 times more efficient at killing than other lysins and chemical disinfectants. Just 10 ng of PlyC kills 10^7^ bacteria in 5 seconds.^16^

“The structure gives us insights into how to engineer other lysins to work better,” Nelson says. He and his colleagues speculate that similar lysins could control methicillin-resistant *S. aureus*- in hospitals and nursing homes, and *Streptococcus pneumoniae* in schools, daycare centers, and military barracks.^16^

Phages are also being explored for use in treating acne. Up to 60% of strains of *Propionibacterium acnes*, the bacterium that causes acne, are antibiotic-resistant, and improved acne therapies are needed. Jenny Kim, a dermatologist at the University of California, Los Angeles, Medical School, suspects that naturally occurring skin phages protect some people from acne. “We all carry *P. acnes*,” she explains, “but not everyone gets acne.” She suggests the skin microbiome of people with clear skin may have phage populations that keep *P. acnes* in check.

Kim and colleagues sequenced the genomes of phages obtained from the sebaceous follicles (where *P. acnes* concentrates) of people with and without acne. They identified a variety of phages that kill *P. acnes* to varying degrees.^17^ Kim says there is “a great therapeutic opportunity” to develop a topical phage treatment for acne.

In addition to treating bacterial infections, phages may also help in diagnosing them. A team from Albert Einstein College of Medicine, the University of Pittsburgh, and the Nelson R. Mandela School of Medicine in Durban, South Africa, engineered a fluorophage carrying green fluorescent protein reporter genes that glows when it infects *Mycobacterium tuberculosis*, the cause of tuberculosis (TB).^18^ The goal of developing the fluorophage was to speed the detection of drug-resistant strains of *M. tuberculosis* in sputum from TB patients. Standard cell culture tests take up to two months; meanwhile, just a small number of inhaled bacteria spread TB.

“The technology looks promising and a more reasonable goal than treating TB with phages,” says codeveloper Graham Hatfull, a professor of biotechnology at the University of Pittsburgh, although he says a phage nasal spray could potentially prevent the transmission of TB. “Phages are an extraordinary reservoir for new genes and applications,” Hatfull says.

## Water Treatment

Water treatment is still another area where phages are seeing a renaissance in the United States. Sewage contains up to 1,000 times more viruses than other water bodies.^19^ Civil and environmental engineers screen sewage to find phages that can improve drinking and wastewater treatment. Briefly, they pass wastewater samples through nylon filters and collect the filtrate. Phages in the filtrate are grown on agar plates seeded with the bacterium the engineers want to kill. After this first round of killing, the phage solution is collected and centrifuged; the supernatant liquid is a rich source of the desired phages.^20^ “Phages are a new area of research for wastewater treatment, and they could easily integrate into existing systems,” says Ramesh Goel, an associate professor of civil and environmental engineering at the University of Utah in Salt Lake City.

During activated sludge processing of sewage, sludge settles in tanks, and the supernatant is drained off for further purification. But this process is foiled by filamentous microbes such as *Sphaerotilus natans*, which grow long tentacles that suspend sludge and impede settling. Disinfectants such as chlorine are added to kill these bacteria, but they tend to kill bacteria near the water’s surface, and there are plenty more below that quickly take over when treatment stops.

In experiments Goel has targeted these problematic filamentous bacteria with phages isolated from sewage. In one study turbid wastewater contaminated with *S. natans* showed reduced sludge volume and clearer supernatant after 12 hours; in addition, the phages remained stable and active for more than 9 months and tolerated temperature and pH fluctuations common to activated sludge processes.^21^

Zhiqiang Hu, an associate professor of civil and environmental engineering at the University of Missouri, Columbia, has conducted similar studies with bacterial biofilms of *P. aeruginosa*, which commonly clog filters at drinking water treatment plants and require chlorine and expensive flushing to clean them. Hu isolated phages from sewage that kill *P. aeroginosa* biofilms and tested them against chlorine, the standard treatment, which removed 40% of *P. aeroginosa* biofilms. Phages alone killed 89%, and phages followed by chlorine knocked out 97% of the biofilms.^20^

As in many natural settings—including the human body—wastewater treatment plants maintain a careful balance of microbes, with many desirable species breaking down wastes and controlling odors at the plants.^20^ “The goal is to remove pathogenic bacteria with minimal impact on beneficial bacteria,” says Hu. Because the number of phages surrounding water treatment facilities is huge, “adding desired phages should not cause environ-mental or health concerns,” he adds.

## Pros and Cons

Although bacteria do develop resistance to their viral predators, several mutations must occur to beat all the phages in a cocktail.^22^ “The chance of bacteria becoming resistant to multiple phages in a cocktail simultaneously is very unlikely,” says Sharma. “Using multiple phages targeting the same pathogen in a cocktail provides a built-in contingency against development of phage resistance in bacteria.”

Sulakvelidze predicts that sometime in the future his company’s products will need to be updated with new phages. Should resistance occur, he says new phages will be identified and added to formulations to restore potency.

But while phages offer unprecedented flexibility to address bacterial resistance, phage cocktails require that large amounts of the viral workhorses be grown inside pathogenic bacteria in a laboratory, theoretically putting workers and the environment at risk. Taking advantage of lysins offers a safer alternative, according to Seal.

**Figure:**
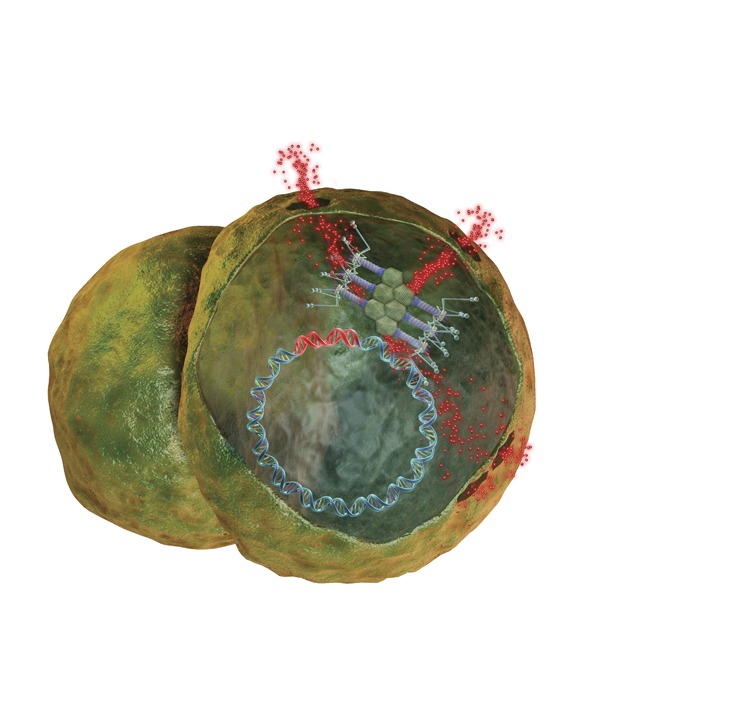
A biofilm of *Staphylococcus* on the inner surface of a needleless intravenous connector. If phages become common tools in medical applications and other uses, the public may need reassurance that viruses can be helpful, not just harmful. © Science Source

**Education Needed**

Phages are the most ubiquitous microbes on Earth. Our dental plaque, gastrointestinal tract, skin, and other organs, as well as our food and drinking water, are loaded with these microorganisms, and we live harmoniously with them.^22^ But if phages become more commonly used in health care and other settings, people may be skeptical or even frightened to learn they are actually viruses. “The public perceives all viruses as dangerous,” says Zhiqiang Hu of the University of Missouri, Columbia—they think of influenza, polio, and AIDS.

Sources interviewed for this story agree on the need for education to ease the acceptance of phage therapies by the general public. “Initially the idea may sound scary, but if phages work well, the public will accept them,” predicts UCLA’s Jenny Kim. She points out that using viruses to kill bacteria is similar to using live vaccines to prevent disease. And many people now embrace the idea of “good bacteria” and purchase probiotic foods like yogurt.

“Phages fit into this progressively appreciated trend that not all microbes are bad, and many are beneficial,” says Intralytix chief scientific officer Alexander Sulakvelidze. “Phages, if used properly, can have a tremendous positive impact on many aspects of our lives, from food safety to human health.”

Two hands-on educational programs are familiarizing students with phages. Graham Hatfull of the University of Pittsburgh spearheaded the Phage Hunters Integrating Research and Education (PHIRE) program. Local high-school and university students collect soil and isolate phages from it, purify and extract phage DNA, and sequence it at the Pittsburgh Bacteriophage Institute’s genomic center. Then they use bioinformatics tools to analyze and annotate the genomes.^28^^,^^29^

PHIRE students have discovered and named 1,000 new phages, and Hatfull says the students are keenly aware that they are advancing the science through their work in the program. PHIRE gave rise to the national Science Education Alliance Phage Hunters Advancing Genomics and Evolutionary Science (SEA-PHAGES) project, a two-term, research-based curriculum for college freshman. More than 70 universities and 2,000 students are involved yearly in SEA-PHAGES. The Howard Hughes Medical Institute funds both programs.^30^

Professional phage hunters can keep up on the latest research through a new journal named Bacteriophage, the first journal dedicated to all aspects of phage research (Sulakvelidze is the editor-in-chief).31 Topics covered in the international, peer-reviewed journal include basic biology and taxonomy of phages, bacteriophage–host cell interactions, practical applications of phages, and results of clinical trials.^32^

But lysins have their limitations, too. They work best on Gram-positive bacteria (e.g., *Streptococcus*, *Staphylococcus*, *Clostridium*). Although phages have proven effective against Gram-negative bacteria (e.g., *Salmonella*, *E. coli*, *Pseudomonas*), the outer fatty membrane protecting these types of bacteria hinders penetration by lysins alone.^18^ And like all therapeutic proteins, lysins are more expensive to develop and manufacture than phages, according to both Seal and Nelson. Higher costs could make lysins impractical for some industrial purposes.

Meanwhile, gaining approval for human phage therapeutics presents unique chal-lenges of its own. When Intralytix sought FDA approval for its ListShield product “there were zero guidelines for us to follow, and it took four years,” says Sulakvelidze. Approval of the company’s second product went more smoothly, and its approval took about 1.5 years. The process was “mutually educational for us and the FDA, and we hope we cleared the way for others to follow,” Sulakvelidze says. The FDA is finalizing guidelines for other phage preparations.^22^

Human phage therapies are regulated as drugs and biological products, and require an Investigational New Drug (IND) Application for testing in people. For traditional drugs, the FDA wants each component of a drug combination to be proven safe and effective both individually and in combination. For phage cocktails, that means the activity, potency, and stability of each phage must be demonstrated, according to FDA spokeswoman Rita Chappelle. The logistical challenges posed by this requirement may be a roadblock for phage therapy.^22^

“FDA encourages manufacturers interested in conducting clinical studies of phages for use in addressing human diseases or conditions to contact the Agency as soon as possible during product development,” Chappelle says, adding that developers of phage therapeutics can request a pre-IND meeting.

DNA sequencing, proteomic analysis, cytotoxicity testing, and transmission electron microscopy are helping to characterize human phage preparations. These tools confirm the desired lytic process and the absence of toxins and impurities, and help to establish standards for good manufacturing practices for clinical testing.^23^

It will take time to sort out the advan-tages and disadvantages of phages as well as the logistics of registering them with appropriate authorities. But phages’ proponents insist the benefits are worth it: Whether applied as cocktails, lysins, or individual phages, these agents offer the potential of a vast arsenal of new antibacterial agents. Says Hatfull, “There’s much more to be done and lots of opportunities to develop phages.”
